# Incidence of HIV and the Prevalence of HIV, Hepatitis B and Syphilis among Youths in Maputo, Mozambique: A Cohort Study

**DOI:** 10.1371/journal.pone.0121452

**Published:** 2015-03-23

**Authors:** Edna Omar Viegas, Nelson Tembe, Eulália Macovela, Emília Gonçalves, Orvalho Augusto, Nália Ismael, Nádia Sitoe, Caroline De Schacht, Nilesh Bhatt, Bindiya Meggi, Carolina Araujo, Eric Sandström, Gunnel Biberfeld, Charlotta Nilsson, Sören Andersson, Ilesh Jani, Nafissa Osman

**Affiliations:** 1 Instituto Nacional de Saúde, Maputo, Mozambique; 2 Department of Laboratory Medicine, Karolinska Institutet, Huddinge, Sweden; 3 Faculdade de Medicina, Universidade Eduardo Mondlane, Maputo, Mozambique; 4 Hospital Central de Maputo, Maputo, Mozambique; 5 Public Health Agency of Sweden, Solna, Sweden; 6 Department of Microbiology, Tumor and Cell Biology, Karolinska Institutet, Stockholm, Sweden; 7 Elizabeth Glaser Pediatric AIDS Foundation, Maputo, Mozambique; 8 Department of Education and Clinical Research, Karolinska Institutet, Södersjukhuset, Stockholm, Sweden; 9 Department of Laboratory Medicine, Faculty of Medicine and Health, Örebro University, Örebro, Sweden; University of Cincinnati College of Medicine, UNITED STATES

## Abstract

**Background:**

Prevalence of HIV in Mozambique among individuals aged 15–49 years is 11.5%. The HIV prevalence is higher in women than in men across the country, peaking at ages 25–29 years and 35–39 years, respectively. In this study, we aimed at determining the prevalence and incidence of HIV, prevalence of Hepatitis B (HBV), and prevalence of syphilis in youths. We also characterized a cohort of youths for future participation in phase I/II HIV vaccine trials.

**Methods:**

The study was conducted at a youth clinic in Maputo Central Hospital from August 2009 to October 2011. Youths of both genders aged 18–24 years (n = 1380) were screened for HIV using a sequential algorithm of two immunochromatographic assays, HBV using an enzyme linked immunosorbant test, and syphilis using a treponemal immunochromatographic strip test. The HIV seronegative participants (n = 1309) were followed-up for 12 months with quarterly study visits. The clinical and behavioral data were collected using structured questionnaires. The HIV seroconversions were confirmed by a molecular assay.

**Results:**

The study population was female dominant (76.8%). All participants had a formal education, with 44.6% studying for technical or higher education degrees. The mean age at sexual debut was 16.6 years (SD: ±1.74), with 85.6% reporting more than one sexual partner in life. The screening showed the prevalence of HIV, HBV, and syphilis at 5.1% (95% CI: 3.97–6.31), 12.2% (95% CI 10.5%–14.0%), and 0.36% (95% CI 0.15%–0.84%), respectively. The HIV incidence rate was found to be 1.14/100 person years (95% CI: 0.67–1.92). Retention rates were stable throughout the study being 85.1% at the last visit.

**Conclusion:**

Incidence of HIV in this cohort of youths in Maputo was relatively low. Also, the prevalence of HIV and syphilis was lower than the national values in this age group. However, the HBV prevalence was higher than in previous reports in the country.

## Introduction

HIV/AIDS continues to cause high morbidity and mortality, particularly in sub-Saharan Africa [1]. In 2012, 35.3 million people were infected by HIV worldwide. Mozambique has the fifth highest prevalence of HIV in the world, with 11.5% of the 15–49 years old population infected with HIV [2,3]. Women have a higher prevalence than men (13.1% vs 9.2%) [3]. In Mozambique, the peak of HIV prevalence is found in women aged 25–29 years (16.8%) and in men aged 35–39 years (14.2%) [3]. In 2012, the world HIV prevalence in youths aged 15–24 was 0.8% and 4.7% in Sub-Saharan Africa [4]. In Mozambique, the prevalence in this age group, in 2009, was 4.2% (4.8% and 3.5% in women and men, respectively) [3]. In sub-Saharan Africa, women face significantly higher risk of HIV acquisition and are infected at earlier ages. Factors contributing to a higher rate of HIV infection in women include gender inequalities leading to unequal power relationships, unequal access to education and economic opportunities, [1] and biological factors.

Sexually transmitted infections (STIs), including hepatitis B and syphilis, are important public health concerns and constitute risk factors for HIV acquisition [5]. Worldwide, it is estimated that more than two billion people have been infected with the hepatitis B virus (HBV) [6], a disease that can lead to chronic infection, development of liver cirrhosis and hepatocellular carcinoma. Sub-Saharan Africa is considered by the World Health Organization (WHO) as a highly endemic area for HBV [7]. Transmission of HBV in sub-Saharan Africa commonly occurs during childhood. Other modes of transmission such as sexual and parenteral are also important, particularly in areas where unprotected sex is a common practice. In Mozambique, studies among blood donors demonstrate that HBV prevalence in the northern and southern regions of the country is 10.6% [8] and 9.3% [9], respectively. Syphilis is an important cause of morbidity and mortality, especially in pregnant women and infants [10]. In Mozambique, the national prevalence of syphilis in pregnant women in 2011 was 2.2%, ranging from 1.2% to 8.2% [11] in the southern and northern region, respectively.

Youth clinics (“SAAJ, Serviço Amigo do Adolescente e Jovem”) have been established throughout Mozambique with the aim of providing sexual and reproductive health services, and to encourage behavior change through peer education. A study conducted by Melo et al. [12] in 2002–2003 demonstrated that youths attending a youth clinic in Maputo had high level of awareness of STIs including HIV, and that the HIV prevalence in this group was lower than the estimated prevalence in the general population [3]. Behavioral change is one of the targets of the National Aids Council of Mozambique [13]. However, from 2003 to 2009, disturbing signs of increasing sexual risk behavior among young Mozambicans aged 15–24 years have emerged [1]. Although development of a safe, effective, and affordable preventive vaccine is far in the horizons, it may be the best long-term hope to control the HIV/AIDS pandemic in resource-limited settings [14].

In order to understand the dynamics of HIV transmission, data on new infections is needed [15,16]. This is particularly important in the age group where most of the transmissions are occurring. Mozambique has an expansive age pyramid, i.e., the majority of the population in the country is young. Approximately one third of the population is within the age group of 15–49 years [17], where the highest rate of HIV infections is occurring. Although HIV prevalence is well documented in Mozambique, information on HIV incidence in young population is lacking. We have studied a cohort of young people at a clinic providing services to adolescents and youths (“SAAJ”-clinic) in Maputo. The aims of the present study were to: 1) describe the socio-behavioral characteristics of this population, 2) determine the prevalence and incidence of HIV infection, 3) determine the prevalence of hepatitis B and syphilis, and 4) assess the suitability of the cohort for possible participation in phase I/II HIV vaccine trials.

## Subjects and Methods

### Ethics statement

This study was approved by the National Health Bioethics Committee of Mozambique (reference 148/CNBS on May 8, 2009) and followed the GCP ICH guidelines. Written informed consent was obtained from each participant prior to conducting any study procedures.

### Study population

This prospective cohort study was conducted at the Maputo Central Hospital, Mozambique, between August 2009 and October 2011. The participants were recruited and followed-up at an outpatient youth clinic specializing in provision of sexual and reproductive health services including STI/HIV prevention and care. Youths aged 18–24 of both genders and residing in Maputo were invited to participate in the study. Information sheets were handed to those who showed interest in taking part in the study. Trained research staff conducted individual detailed review of study procedures. All questions regarding study participation were addressed prior to signing the informed consent.

### Screening and enrolment

At baseline, a face-to-face interview to assess knowledge, attitude and practices (KAP survey) in relation to HIV and STIs was conducted by trained peer counselors using structured questionnaires. The socio-demographic data and clinical history were obtained by the study nurses who also performed physical examination on all the study participants. Screening for HIV, HBV and syphilis was done before enrolment and dried blood spots (DBS) were collected and stored. Pre- and post-HIV test counseling was offered individually and confidentially according to the national guidelines. Male and female condoms were provided to all the participants. Participants with negative or indeterminate HIV test results were enrolled in the longitudinal HIV incidence study, which began on the same day. Locator information such as telephone numbers and residential address were also collected by the study team.

### Follow-up visits

Upon enrolment, participants were asked to return to the study site on a quarterly basis over the course of 12 months, i.e., for three follow-up visits. The visit window was determined to be +/−8 weeks. At each follow-up visit the participants underwent: 1) a one-to-one interview to assess the HIV-related risk behaviors using a structured questionnaire, 2) clinical examination to identify possible signs and symptoms of acute HIV infection, 3) HIV counseling and testing, and 4) STIs risk reduction counseling. Male and female condoms were provided at all study visits. At each visit, DBS were collected and stored at the study laboratory for the confirmation of time to event, i.e., time between a negative and positive HIV result. Contact details and locator information were updated at each follow-up visit. Participants failing to attend a scheduled visit were contacted by phone the following day. Active tracing was done whenever phone contact was not successful. A loss to follow-up was defined as a participant who did not attend the remaining follow-up study visits and was not reachable by phone or active tracing. Participants who expressed a desire to discontinue their participation in the study were included under the category of study discontinuation. Seroconverting subjects were defined as those who presented with a non-reactive HIV rapid test on a study visit followed by a reactive result on the subsequent visit.

### Laboratory testing

HIV testing followed the national algorithm [18]. Youths were tested using an on-site sequential algorithm of two immunochromatographic assays: the Determine HIV-1/2 (Abbott Laboratories, Illinois, USA), followed by a confirmatory test UniGold HIV-1/2 (Trinity Biotech, Bray, Wicklow, Ireland). An individual was considered HIV infected when both assays were reactive. An indeterminate HIV test result was defined as a reactive Determine test followed by a non-reactive UniGold assay. As a means of determining the timing of HIV infection in a subject with a reactive HIV rapid assay on a follow-up visit, the DBS samples collected on Whatman filter papers from the previous visits were tested using a molecular assay (Roche Amplicor HIV-1 DNA test, version 1.5, Roche Molecular Diagnostics, Branchburg, NJ) [19].

HIV-1 viral load was measured using a COBAS Taqman48 analyzer (Roche Molecular Diagnostics, Mannheim, Germany), and CD4+ T-cells count was determined using a Becton Dickinson FACSCalibur instrument (Biosciences Corp, NJ, USA). Both tests were performed in all HIV infected participants on the day of diagnosis.

Serum samples were collected for the hepatitis B surface antigen (HBsAg) screening. Tests were performed using an enzyme linked immunosorbent assay (HUMAN GmbH, Wiesbaden, Germany). Syphilis testing was performed on-site, in whole blood, using a treponemal immunochromatographic strip test (SD BIOLINE Syphilis 3.0, Standard Diagnostics, Kyonggi-do, Korea).

### HIV/Syphilis treatment and Hepatitis B referral

Participants diagnosed with an HIV infection during the study received HIV care and treatment services at the youth clinic. The CD4+ T-cells count and the HIV-1 viral load were determined in each incident case and made available for clinical follow-up. Participants with a reactive syphilis test were treated at the study site per national treatment guidelines and those diagnosed with HBV infection where referred for clinical management at the Gastroenterology Department of Maputo Central Hospital.

### Data processing and statistical analysis

Data was entered in a MySQL database version 5.1 with a frontend designed in Microsoft Office Access 2007. Validating rules and skipping patterns were implemented to ensure data quality. Data was exported to Stata 12 (StataCorp 2011, Stata Statistical Software: Release 12, College Station, TX: StataCorp LP) for statistical analyses. Descriptive statistics were used to summarize the baseline demographic and behavioral characteristics. Categorical variables were expressed in percentages, and continuous data as means with respective standard deviations (SD). The significance level was set at 5%. For baseline data, the unadjusted odds ratios and their 95% confidence intervals for each cofactor were calculated. Cofactors with a p-value less than 0.2 on either a Pearson or Fisher’s exact chi-square were included on the logistic multivariate analysis using a stepwise backward regression. At each step, variables were tested to be removed from the model at a significance level of 0.10 using the likelihood-ratio test. Two models were built, one for overall participants, and one per gender.

The HIV incidence rate (IR) was calculated by dividing the number of new HIV cases by the person-years (PY) of the cohort. Uninfected participants that attended only one study visit (visit 1) were considered censored at the 60^th^ day of follow-up. The IR was expressed as number of cases per 100 PY of follow-up, and its 95% exact Poisson confidence intervals (CI) were reported. Hazard rates and their robust confidence intervals were reported for each cofactor. The retention rates per visit were calculated by dividing the number of participants who attended a study visit by the expected number, and was expressed in percentages. HIV seroconversions were excluded from the denominator for the following visit.

## Results

### Baseline demographic and behavioral characteristics

A total of 1380 youths were enrolled in the HIV prevalence study, out of which 320 (23.2%) were males and 1060 (76.8%) were females. The demographic characteristics of all screened participants are presented in Table 1. The mean age of the participants was 20.9 years (SD: ±1.71) with males being older than females (21.4 vs 20.7, p<0.001). Almost all the participants (98.6%) were single, and approximately half (55.4%) of them had primary or secondary education. There were no illiterate participants.

**Table 1:** Baseline socio-demographic and behavioral characteristics.

**Characteristic**	**Female**		**Male**		**Total**		**p**
	**N**	**%**	**N**	**%**	**N**	**%**	
Total Screened	1060		320		1380		
Marital Status							
Single	1043	98.4%	318	99.4%	1361	98.6%	0.274*
Married/Cohabitating	17	1.6%	2	0.6%	19	1.4%	
Age [mean (SD)]	20.7 (1.70)		21.4 (1.64)		20.9 (1.71)		**< 0.001**
Education							
Primary and Secondary	615	58.0%	149	46.6%	764	55.4%	**< 0.001**
Technical training	260	24.5%	78	24.4%	338	24.5%	
University degree	185	17.5%	93	29.1%	278	20.1%	
Occupation							
Student	1030	97.2%	299	93.4%	1329	96.3%	**0.002**
Employed	30	2.8%	21	6.6%	51	3.7%	
Religion							
Christian	961	90.7%	285	89.1%	1246	90.3%	0.398
Other	99	9.3%	35	10.9%	134	9.7%	
Age at sexual debut (years)							
Mean (SD)	16.8 (1.55)		16.0 (2.16)		16.6 (1.74)		**< 0.001**
Less than 18	715	67.5%	235	73.4%	950	68.8%	**0.043**
18 or more	345	32.5%	85	26.6%	430	31.2%	
Number of sex partners in life							
1	184	17.4%	14	4.4%	198	14.3%	**< 0.001**
> 1	876	82.6%	306	95.6%	1182	85.7%	
Number of sex partners in the last 6 months							
0–1	914	86.2%	196	61.3%	1110	80.4%	**< 0.001**
> 1	146	13.8%	124	38.8%	270	19.6%	
Condom use in the last sexual intercourse							
No	390	36.8%	92	28.8%	482	34.9%	**0.008**
Yes	670	63.2%	228	71.3%	898	65.1%	
Had a STI before							
No	710	67.0%	263	82.2%	973	70.5%	**< 0.001**
Yes	350	33.0%	57	17.8%	407	29.5%	
Alcohol consumption							
No	593	55.9%	99	30.9%	692	50.1%	**< 0.001**
Yes	467	44.1%	221	69.1%	688	49.9%	
Drug use							
No	1059	99.9%	314	98.1%	1373	99.5%	**0.001** *
Yes	1	0.1%	6	1.9%	7	0.5%	

* Fisher's exact chi-squared test.

Sexual behavior characteristics at screening are listed in Table 1. The mean age at sexual debut was 16.6 years (SD: ±1.74). Male participants reported to have initiated sexual activity earlier than female participants, 16.0 years (SD: ±2.16) vs 16.8 (SD±1.55), respectively, p<0.001, with 68.8% of youths reporting to have initiated sexual activity before the age of 18 years. A total of 1182 (85.7%) participants reported to have had more than one sexual partner in life, and 270 (19.6%) reported multiple sex partners six months prior to the study participation. The number of participants that reported more than one sexual partner in the previous six months was significantly higher in male than in female participants (38.8% vs 13.8%, p<0.001). About one third of youths (29.5%) reported at least one episode of STI in life and 86.0% of them were females (p<0.001). Use of condom during the last reported sexual intercourse was significantly different between genders (p = 0.008), being 63.2% for females and 71.3% for males. Approximately, half of the study participants (49.9%) reported use of alcohol (69.1% vs 44.1% in men and women, respectively, p<0.001), while use of tobacco, injectable drugs and/or other drugs was reported only by a few (3.44% vs 0.94% in men and women, respectively) (data not shown).

### HIV prevalence

The overall HIV prevalence at the time of screening was 5.1% (71 infections; 95%CI: 3.97–6.31; Table 2), and the prevalence was significantly higher in women than in men (5.8% vs 3.1%, p = 0.018). Data on HIV prevalence by gender in relation to socio-demographic and behavioral aspects are presented in supplementary S1 Table and S2 Table. For each year of age, the odds to be HIV-infected increased by 81% in men (p = 0.020) and by 37% in women (p<0.001). Although the HIV infections were more frequent (73%) in youths with lower educational level, analysis by gender demonstrated that the impact of education was only significant in the female population (p = 0.006). Sexual debut at age below 18 was associated with higher HIV prevalence in females (p = 0.005). No association was found between HIV infection and the number of sexual partners in life or with the number of sexual partners six months prior to the study participation. Men who reported to have had sexually transmitted infections had a significantly higher HIV prevalence (p = 0.033). This was not observed in the female population.

**Table 2:** Baseline socio-demographic and behavioral characteristics and HIV prevalence.

**Characteristic**	**Total**		**HIV negative**		**HIV positive**			**Unadjusted**			**Adjusted**		
	**N**	**%**	**N**	**%**	**N**	**%**	**Prevalence**	**OR**	**CI 95%**	**p**	**OR**	**CI 95%**	**p**
Total Screened	1380		1309		71		5.1%						
Gender													
Male	320	23.2%	310	23.7%	10	14.1%	3.1%	0.53	0.27–1.04	0.066	0.43	0.21–0.87	**0.018**
Female	1060	76.8%	999	76.3%	61	85.9%	5.8%	-	-		-	-	-
Age (change in a year)	-	-	-	-	-	-	-	1.29	1.12–1.48	**< 0.001** *	1.43	1.23–1.65	**< 0.001**
Marital Status													
Single	1361	98.6%	1291	98.6%	70	98.6%	5.1%	-	-	1.000†	-	-	-
Married/Cohabitating	19	1.4%	18	1.4%	1	1.4%	5.3%	1.02	0.13–7.79		-	-	-
Education													
Primary and Secondary	764	55.4%	712	54.4%	52	73.2%	6.8%	-	-	**0.005**	-	-	-
Technical training	278	20.1%	267	20.4%	11	15.5%	4.0%	0.33	0.16–0.71		0.55	0.28–1.08	0.084
University degree	338	24.5%	330	25.2%	8	11.3%	2.4%	0.56	0.29–1.10		0.29	0.14–0.62	**0.002**
Occupation													
Student	1329	96.3%	1261	96.3%	68	95.8%	5.1%	-	-	0.743†	-	-	-
Employed	51	3.7%	48	3.7%	3	4.2%	5.9%	1.16	0.35–3.82		-	-	-
Religion													
Christian	1246	90.3%	1185	90.5%	61	85.9%	4.9%	0.64	0.32–1.28	0.205	-	-	-
Other	134	9.7%	124	9.5%	10	14.1%	7.5%	-	-		-	-	-
Age at sexual debut (years)													
Less than 18	950	68.8%	894	68.3%	56	78.9%	5.9%	-		0.064	-	-	-
18 or more	430	31.2%	415	31.7%	15	21.1%	3.5%	0.58	0.32–1.03		0.45	0.25–0.82	**0.009**
Number of sex partners in life													
1	198	14.3%	190	14.5%	8	11.3%	4.0%	-		0.449			
> 1	1182	85.7%	1119	85.5%	63	88.7%	5.3%	1.34	0.63–2.84		-	-	-
Number of sex partners in the last 6 months													
0–1	1110	80.4%	1050	80.2%	60	84.5%	5.4%	-		0.376			
> 1	270	19.6%	259	19.8%	11	15.5%	4.1%	0.74	0.39–1.43		-	-	-
Condom use in the last sexual intercourse													
No	482	34.9%	456	34.8%	26	36.6%	5.4%	-	-	0.759	-	-	-
Yes	898	65.1%	853	65.2%	45	63.4%	5.0%	0.93	0.56–1.52		-	-	-
Alcohol consumption													
No	692	50.1%	659	50.3%	33	46.5%	4.8%	-	-	0.526	-	-	
Yes	688	49.9%	650	49.7%	38	53.5%	5.5%	1.17	0.72–1.88		-	-	-
Drug use													
No	1373	99.5%	1302	99.5%	71	100.0%	5.2%	-	-		-	-	-
Yes	7	0.5%	7	0.5%	0	0.0%	0.0%	-	-		-	-	-
Had a STI before													
No	973	70.5%	927	70.8%	46	64.8%	4.7%	-	-	0.281	-	-	-
Yes	407	29.5%	382	29.2%	25	35.2%	6.1%	1.32	0.80–2.18		-	-	-

* Likelihood Chi-squared test

† Fisher's exact chi-squared test

### Participant retention and HIV incidence

**Fig 1:**
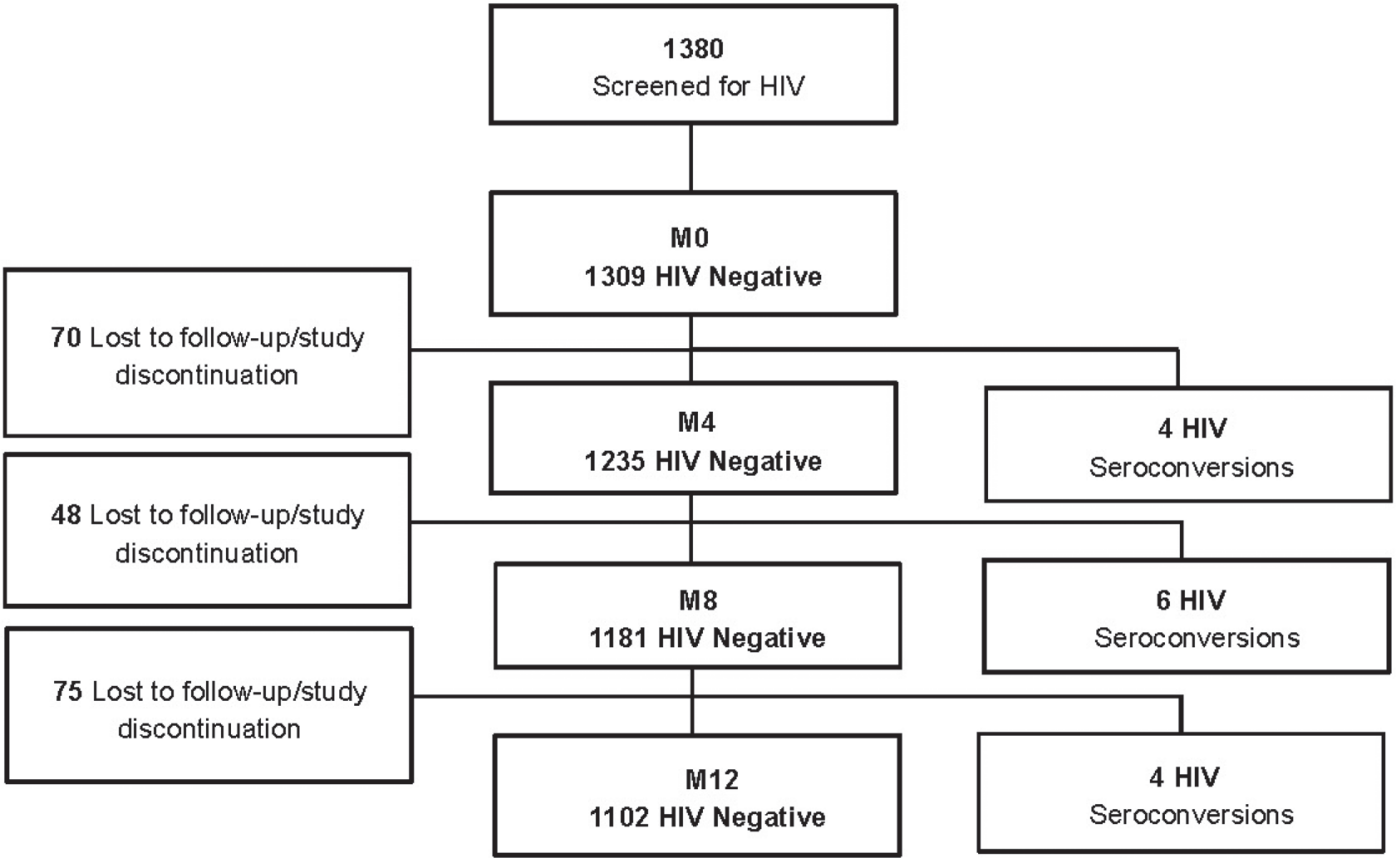
Flow of participants during the study. M, Month.

Participants enrolled in the HIV incidence study (n = 1309) attended a total of 3414 follow-up visits, which accounted for a total of 1229.78 person years of follow-up. The average years of follow-up were 0.94 (range 0.16–1.17). Fourteen seroconversions occurred throughout the study, four within the first four months of the study initiation, six between the fifth and eighth month, and four within the last four months of study follow-up (Fig. 1). The HIV incidence rate was 1.14/100PY (95% CI: 0.67–1.92). All incident infections occurred in the female participants, and the HIV incidence in women was 1.49/100 Women Years (WY) (95%CI: 0.88–2.51). None of the seroconverters had hepatitis B or syphilis at the time of enrolment. There were no associations between gender, age, level of education, religion or sexual behavior and increased risk of HIV acquisition (Table 3). We observed a non-significant trend towards higher risk of HIV acquisition in participants who were married or cohabiting (p = 0.055). The cohort retention was 82.2% in the first quarter, 81.1% in the second quarter and 85.1% in last quarter of the study. Table 4 shows the compliance rates in relation with the socio-demographic characteristics. Approximately two-thirds (73.5%) of the participants completed the study schedule and attended all four study visits. Single and higher educated participants were significantly more compliant than those who were married and less educated (p = 0.049 and p = 0.043, respectively). Students had a better visit compliance rate than those with a formal employment (p = 0.04).

**Table 3:** Risk factors for acquisition of HIV infection during subsequent follow-ups of uninfected volunteers.

**Characteristic**	**Total**		**Person-Years**		**Incidence Rates**			**Hazard Rates (HR)**		
	**N**	**%**	**N**	**%**	**N**	**IR per 100PY**	**CI 95%**	**HR per 100PY**	**CI 95%**	**p**
Total Screened	1309		1229.8		14	1.14	0.62–1.91			
Gender										
Male	310	23.7%	289.56	23.5%	0	0.00	0.00–1.27*	-	-	-
Female	999	76.3%	940.23	76.5%	14	1.49	0.81–2.50	-	> 1.02**	**0.023** **
Age (per one year increase)	1309	-	1229.8	-	-	-	-	1.06	0.82–1.39	0.647
Marital Status										
Single	1291	98.6%	1215.07	98.8%	13	1.07	0.57–1.83	-	-	-
Married/Cohabitating	18	1.4%	14.71	1.2%	1	6.80	0.17–37.88	5.61	0.97–32.62	0.055
Education										
Primary and Secondary	712	54.4%	658.95	53.6%	9	1.37	0.62–2.59	-	-	-
Technical training	330	25.2%	318.56	25.9%	2	0.63	0.08–2.26	0.47	0.10–2.16	0.330
University degree	267	20.4%	252.28	20.5%	3	1.19	0.25–3.48	0.90	0.24–3.30	0.872
Occupation										
Student	1261	96.3%	1184.4	96.3%	13	1.10	0.58–1.88	-	-	-
Employed	48	3.7%	45.37	3.7%	1	2.20	0.06–12.28	1.82	0.27–12.65	0.524
Religion										
Christian	1185	90.5%	1111.3	90.4%	12	1.08	0.55–1.88	-	-	-
Other	124	9.5%	118.50	9.6%	2	1.69	0.20–6.10	1.42	0.31–6.43	0.648
Age at sexual debut (years)										
Less than 18	894	68.3%	841.45	68.4%	10	1.19	0.64–2.21	-	-	-
18 or more	415	31.7%	388.34	31.6%	4	1.03	0.39–2.74	0.86	0.27–2.74	0.802
Had sex in the last 4 months†										
No	221	-	79.47	6.5%	0	0.00	0.00–4.64*	-	-	-
Yes	3075	-	1138.8	92.6%	14	1.23	0.67–2.06	-	> 0.23**	0.389**
Had sex with sex worker										
No	3264	-	1204.3	97.9%	14	1.16	0.64–1.95	-	-	-
Yes	31	-	13.44	1.1%	0	0.00	0.00–27.4*	-	0–27.02**	0.856**
Had a STI before										
No	927	70.8%	868.28	70.6%	8	0.92	0.40–1.82	-	-	-
Yes	382	29.2%	361.51	29.4%	6	1.66	0.61–3.61	1.76	0.60–5.16	0.302
Alcohol consumption										
No	659	50.3%	622.89	50.7%	6	0.96	0.35–2.10	-	-	-
Yes	650	49.7%	606.90	49.4%	8	1.32	0.57–2.60	1.33	0.47–3.79	0.592
Drug use										
No	1302	99.5%	1223.1	99.5%	14	1.14	0.63–1.92	-	-	-
Yes	7	0.5%	6.65	0.5%	0	0.00	0.00–0.55*	-	0–55.44**	0.927**

* Unilateral confidence interval

† Collected per visit

** Exact incidence rate ratio or p-value

**Table 4:** Visit compliance rates and factors associated with study retention.

**Characteristic**	**Total Completed visits**								**Total**	**P**
	**One**		**Two**		**Three**		**Four**			
	**N**	**%**	**N**	**%**	**N**	**%**	**N**	**%**	**N**	
Total	70	5.3%	94	7.2%	183	14.0%	962	73.5%	1309	
Gender										
Male	22	7.1%	24	7.7%	41	13.2%	223	71.9%	310	0.423
Female	48	4.8%	70	7.0%	142	14.2%	739	74.0%	999	
Age categories (years)										
< 21	42	5.8%	49	6.8%	98	13.6%	531	73.8%	720	0.755
21 +	28	4.8%	45	7.6%	85	14.4%	431	73.2%	589	
Education										
Primary and Secondary	47	6.6%	59	8.3%	107	15.0%	499	70.1%	712	**0.043**
Technical training	12	4.5%	19	7.1%	36	13.5%	200	74.9%	267	
University degree	11	3.3%	16	4.8%	40	12.1%	263	79.7%	330	
Marital Status										
Single	67	5.2%	91	7.0%	181	14.0%	952	73.7%	1291	**0.049** *
Married/Cohabitating	3	16.7%	3	16.7%	2	11.1%	10	55.6%	18	
Occupation										
Student	68	5.4%	85	6.7%	178	14.1%	930	73.8%	1261	**0.040** *
Employed	2	4.2%	9	18.8%	5	10.4%	32	66.7%	48	
Religion										
Christian	62	5.2%	86	7.3%	163	13.8%	874	73.8%	1185	0.805
Other	8	6.5%	8	6.5%	20	16.1%	88	71.0%	124	

* Fisher's exact chi-square test p-value

### Immunological and virological characteristics of the HIV incident cases

CD4+ T-cells and viral loads were measured in all HIV seroconverters. The median CD4+ T-cell count and viral load were 608 cells/mm^3^ (IQR 397–648) and 74,182 copies/mL (IQR 22,555–166,825), respectively (data not shown).

### Hepatitis B and Syphilis prevalence

Hepatitis B surface antigen was detected in 168 out of 1377 youths (12.2%; 95%CI: 10.5%–14.0%). The number of HBV infections was significantly higher in men (51, 15.9%) than in women (117, 11.1%) (p = 0.02). Among the individuals with HBV infection, eight were HIV positive (4.9%).

Out of 1378 participants, five (0.36%; 95%CI: 0.15%–0.84%) had a positive syphilis test (three female, two male). One individual infected with syphilis was co-infected with HIV, two co-infected with HBV, and one co-infected with both HIV and HBV.

## Discussion

In this study we have documented the seroprevalence of three sexually transmitted infections (HIV, HBV and syphilis) among youths in Maputo, Mozambique. The HIV prevalence in the present study (5.1%) was lower than the overall prevalence found in young people aged 19–24 years in 2009 in Mozambique (10.9%) during a community-based survey (INSIDA) [3]. An antenatal surveillance round (ANSR) conducted in 2011 demonstrated an overall HIV prevalence of 13.2% in pregnant women aged 15–24 [20]. In semi-rural areas of southern Mozambique, the prevalence of HIV was 23.2% in the age group of 18–27 years old men and women in 2010 [21]. Previous results from Melo et al. [12] have also shown a lower HIV prevalence (4%) in young females aged 15–24 at the same youth clinic.

Both our and Melo et al.´s studies have shown that HIV infections decrease with the increase in level of education, suggesting that education has an important role in HIV prevention [12]. These two studies were conducted at a youth clinic that is centrally located, easily accessible, and where HIV counselling and testing, STI treatment, condom provision and sexual behavior education are available free of charge. This could have also contributed to the lower HIV prevalence documented in both the studies.

Here, we have reported a higher number of HIV infections in women. Similar findings were reported by INSIDA [3] in Mozambique and in other south-east African countries [22,23,24]. The rate of HIV infections was higher with increasing age, with women being infected earlier in life compared to men. These findings were also described elsewhere in Mozambique [3] and southern Africa [25]. The age of sexual debut in women was also identified as a risk for HIV infection, corroborating the findings by Hallet et al. [26].

Although adolescents and youths are more prone to risk behaviors such as alcohol abuse and multiple partners, our study did not show any significant association of alcohol abuse and multiple partners with increased risk of HIV-infection. Men who reported to have had STIs were more at risk for HIV infection, which was not true for women. STIs have been described as a risk factor for HIV infection [27] and the lack of association in women in the present study could be a result of their definition of STIs (normal mucous vaginal discharge could have been interpreted as STI), as well as from lack of probing from the staff conducting the questionnaires.

To the best of our knowledge, this is the first cohort study to assess HIV incidence in youths aged 18–24 in Mozambique. Retention rates in our study were similar to those found in other HIV cohort studies [28] or even higher [29]. These rates were stable throughout the study period showing that efforts from peer counselors and nurses to contact participants and to maintain the participants’ willingness to be involved in the study were successful. The incidence found in this study was 1.14/100PY, which is lower compared to the incidence found in studies in pregnant (4.3/100 WY; 95%CI 0.5–7.2) and post-partum Mozambican women (3.2/100 WY; 95%CI: 2.3–4.5) [30]. Two other cohort studies have shown a high HIV incidence in high risk women aged 18–35 years (4.6/100 WY; 95%CI: 2.7–7.3) [31] and 18–24 years (6.5/100 WY; 95%CI: 4.1–9.9) [32] in southern and central Mozambique, respectively. In South Africa, cohort studies in young women in rural and urban areas reported a high incidence of HIV infection, 6.9/110 WY [28] and 14.8/100 WY, respectively [29]. Previous reports in southern Africa have demonstrated that HIV incidence rates are higher in women than in men [22]. In the present study, no incident HIV infections were identified in male participants. The gender disproportion has to be taken into consideration, since 76.8% of the participants were females.

Immunological and virological follow-up of the newly HIV infected participants showed a higher median CD4+ T-cell count (608 cells/mm^3^) and lower median HIV viral load (74,182 copies/mL) compared to findings by McKellar et al. [33]. In general, healthy women have higher CD4+ T-cell counts compared to men [34,35,36]. In our cohort, at baseline, the median CD4+ T-cell count was 824 cells/mm^3^ (IQR 434–1479) in a subset of 155 female participants, and 713 cells/mm^3^ (IQR 357–1155) in a subset of 102 male participants [35]. The four month interval between study visits could have led to a late diagnosis of acute HIV infection, suggesting that immunological recovery was already taking place.

Our study has demonstrated an overall prevalence of HbsAg of 12.2% which is higher than the previously reported prevalence in the country. In Mozambique previous studies in blood donors have reported a HBsAg prevalence of 10.6% [8], 9.3% [9] and 6.01% [37]. Reports in other African countries have shown prevalence of HBV infection as high as 15.5% [38] and 22% [39]. Transmission of HBV in Africa mainly occurs during childhood [6]. The majority of these youths never received Hepatitis B vaccinations. In Mozambique, Hepatitis B vaccination was introduced in the Expanded Program on Immunization in 2001 [40] and is available, free of charge, for infants. Vaccination boosts are not part of the national vaccination program. Therefore, transmission during childhood cannot be excluded. An important step in the epidemiology of Hepatitis B was the understanding that HBV can also be transmitted by sexual contact [6]. In the present study, 35% of youths reported to not have used a condom at the time of the most recent sexual intercourse. Such behavior may contribute to the higher prevalence of HBV infections in this cohort when compared to blood donors [9,37]. Our findings have showed that men were more affected than women (15.9% vs 11.1%), corroborating with previous findings in blood donors [9]. Co-infection with hepatitis B virus is common among HIV-infected individuals [41,42]. In our study we found that 11.3% of HIV-infected individuals were also infected by HBV. These findings are concordant with other results in sub-Saharan Africa, [41,43] indicating that HIV-infected patients could potentially be infected by HBV and could therefore benefit from HBV screening and vaccination. Our study demonstrates that youths are severely affected by HBV infection, and therefore, vaccination should be considered in early ages (to those who were not vaccinated after birth) and boosts given timely (to those who received primary vaccination).

Our results have shown a lower seroprevalence (0.36%) of syphilis compared to previous studies in the same population (2.3%) [12] and with the national rates in pregnant women in 2011 (2.2%) [20]. WHO reports have shown that in 2010, the prevalence of syphilis among women attending antenatal services in Mozambique was 5.7% [44]. Neighboring countries have also reported a high prevalence [45]. Syphilis screening has been practiced in Mozambique since 1978, but it was only in 1995 that it became a key element of the national health plan [46] particularly focusing on pregnant women. The northern region of Mozambique has a higher prevalence of syphilis compared to the south (8.2% vs 1.2%). The proximity to countries with high prevalence of syphilis in the northern part of Mozambique could have contributed to the higher prevalence in this region. Antenatal epidemiological rounds in Mozambique [11,47] have demonstrated that syphilis rates decrease in women with higher education, which supports the findings in our study (45% of the study population had higher educational degree, above secondary level). Additionally we found that all participants with a reactive syphilis test had other sexually transmitted viral infections such as HIV and/or HBV infections, suggesting that the route of transmission was most probably the same and that infection with one STI is a risk for acquisition of other venereal infections [5].

This study was conducted at a youth clinic in Maputo Central Hospital, the main referral hospital in the country. Educational activities and HIV counselling and testing at this site are very well structured and consistently available. The site is centrally located and easily accessible. Many secondary schools and universities are located in the same district area, thus facilitating access to the site. In Mozambique, sexual educational activities also take place at schools and to some extent at universities. Taking into account that almost all participants in this study were students from the surrounding areas, with potential access to information and education in regards to STIs prevention, it is very likely that this cohort with a lower HIV incidence is not representative of youth in Maputo.

One of the limitations of our study was that our cohort was female dominant, with more than 2/3 of the study population being females. This is a reflection of a gender imbalance in the demand for sexual and reproductive health services. This fact could have contributed to the total failure to detect seroconversion in male participants. Since only antibody tests were used for screening, and not the fourth generation combined assays for HIV antibodies and antigen, it cannot be excluded that a few recently infected cases in diagnostic window phase were missed at the last study visit. Incentives are commonly used in population-based cohort studies, and have been shown to be effective in improving retention rates [48,49]. Our retention rate, although relatively stable, was lower on the 3^rd^ study visit. The fact that no monetary compensation was given to the participants may have influenced the rates of attendance since the study population was primarily students with probable economic restrictions.

## Conclusion

Our study characterized a cohort of youths at an outpatient youth clinic specialized in provision of sexual and reproductive health services in Maputo. With the relatively low incidence of HIV, no significant association of risk behavior with HIV acquisition, and with a stable and adequate retention rate we can conclude that the cohort is suitable for possible recruitment into phase I/II HIV vaccine trials and other biomedical intervention studies. This study confirms the endemicity of HBV infections in Maputo, Mozambique suggesting that vaccination campaigns targeting youths should have an impact on the reduction of new HBV infections.

## Supporting Information

S1 Table Baseline socio-demographic and behavioral characteristics and HIV prevalence in male participants. (DOCX)

S2 Table Baseline socio-demographic and behavioral characteristics and HIV prevalence in female participants. (DOCX)
