# Supplementary material for: Incidence of HIV and the Prevalence of HIV, Hepatitis B and Syphilis among Youths in Maputo, Mozambique: A Cohort Study
Source: PLoS One. 2015 Mar 23;10(3):e0121452. doi: 10.1371/journal.pone.0121452 (PMC4370560; doi:10.1371/journal.pone.0121452)
Supplement: S1 Table — (DOCX) [file pone.0121452.s001.docx]

| **S1 Table - Baseline socio-demographic and behavioral characteristics and HIV prevalence in male participants** | | | | | | | | | | |  | |  |  |  | |
| --- | --- | --- | --- | --- | --- | --- | --- | --- | --- | --- | --- | --- | --- | --- | --- | --- |
|  |  |  |  |  |  |  |  |  |  |  |  | |  |  |  | |
|  | **Characteristic** | **Total** | | **HIV negative** | | **HIV positive** | | | **Unadjusted** | | | | **Adjusted** | | | |
|  |  | **N** | **%** | **N** | **%** | **N** | **%** | **Prevalence** | **OR** | **CI 95%** | | **p** | **OR** | **CI 95%** | | **p** |
|  | Total Screened | 320 |  | 310 |  | 10 |  | 3.1% |  |  | |  |  |  | |  |
|  |  |  |  |  |  |  |  |  |  |  | |  |  |  | |  |
|  | Age (change in a year) | - | - | - | - | - | - | - | 1.89 | 1.12 - 3.08 | | **0.011** | 1.81 | 1.10 - 2.99 | | **0.020** |
|  |  |  |  |  |  |  |  |  |  |  | |  |  |  | |  |
|  | Marital Status |  |  |  |  |  |  |  |  |  | |  |  |  | |  |
|  | Single | 318 | 99.4% | 308 | 99.4% | 10 | 100.0% | 3.1% | - | - | | 1.000† | - | - | | - |
|  | Married/Cohabitating | 2 | 0.6% | 2 | 0.6% | 0 | 0.0% | 0.0% | - | - | |  | - | - | | - |
|  |  |  |  |  |  |  |  |  |  |  | |  |  |  | |  |
|  | Education |  |  |  |  |  |  |  |  |  | |  |  |  | |  |
|  | Primary and Secondary | 149 | 46.6% | 142 | 45.8% | 7 | 70.0% | 4.7% | - | - | | 0.384† | - | - | | - |
|  | Technical training | 78 | 24.4% | 77 | 24.8% | 1 | 10.0% | 1.3% | 0.26 | 0.03 - 2.18 | |  | - | - | | - |
|  | University degree | 93 | 29.1% | 91 | 29.4% | 2 | 20.0% | 2.2% | 0.45 | 0.09 - 2.19 | |  | - | - | | - |
|  |  |  |  |  |  |  |  |  |  |  | |  |  |  | |  |
|  | Occupation |  |  |  |  |  |  |  |  |  | |  |  |  | |  |
|  | Student | 299 | 93.4% | 291 | 93.9% | 8 | 80.0% | 2.7% | - | - | | 0.134† | - | - | | - |
|  | Employed | 21 | 6.6% | 19 | 6.1% | 2 | 20.0% | 9.5% | 3.83 | 0.76 - 19.30 | |  | - | - | | - |
|  |  |  |  |  |  |  |  |  |  |  | |  |  |  | |  |
|  | Religion |  |  |  |  |  |  |  |  |  | |  |  |  | |  |
|  | Christian | 285 | 89.1% | 277 | 89.4% | 8 | 80.0% | 2.8% | 0.48 | 0.10 - 2.34 | | 0.300† | - | - | | - |
|  | Other | 35 | 10.9% | 33 | 10.6% | 2 | 20.0% | 5.7% | - | - | |  | - | - | | - |
|  |  |  |  |  |  |  |  |  |  |  | |  |  |  | |  |
|  | Age at sexual debut (years) |  |  |  |  |  |  |  |  |  | |  |  |  | |  |
|  | Less than 18 | 235 | 73.4% | 228 | 73.5% | 7 | 70.0% | 3.0% | - |  | | 0.729† | - | - | | - |
|  | 18 or more | 85 | 26.6% | 82 | 26.5% | 3 | 30.0% | 3.5% | 1.19 | 0.30 - 4.72 | |  | - | - | | - |
|  |  |  |  |  |  |  |  |  |  |  | |  |  |  | |  |
|  | Number of sex partners in life |  |  |  |  |  |  |  |  |  | |  |  |  | |  |
|  | 1 | 14 | 4.4% | 14 | 4.5% | 0 | 0.0% | 0.0% | - | - | | 1.000† |  |  | |  |
|  | > 1 | 306 | 95.6% | 296 | 95.5% | 10 | 100.0% | 3.3% | - | - | |  | - | - | | - |
|  |  |  |  |  |  |  |  |  |  |  | |  |  |  | |  |
|  | Number of sex partners in the last 6 months |  |  |  |  |  |  |  |  |  | |  |  |  | |  |
|  | 0 - 1 | 196 | 61.3% | 190 | 61.3% | 6 | 60.0% | 3.1% | - |  | | 1.000† |  |  | |  |
|  | > 1 | 124 | 38.8% | 120 | 38.7% | 4 | 40.0% | 3.2% | 1.06 | 0.29 - 3.82 | |  | - | - | | - |
|  |  |  |  |  |  |  |  |  |  |  | |  |  |  | |  |
|  | Condom use in the last sexual intercourse |  |  |  |  |  |  |  |  |  | |  |  |  | |  |
|  | No | 92 | 28.8% | 89 | 28.7% | 3 | 30.0% | 3.3% | - | - | | 1.000† | - | - | | - |
|  | Yes | 228 | 71.3% | 221 | 71.3% | 7 | 70.0% | 3.1% | 0.94 | 0.24 - 3.72 | |  | - | - | | - |
|  |  |  |  |  |  |  |  |  |  |  | |  |  |  | |  |
|  | Alcohol consumption |  |  |  |  |  |  |  |  |  | |  |  |  | |  |
|  | No | 99 | 30.9% | 95 | 30.6% | 4 | 40.0% | 4.0% | - | - | | 0.506† | - | - | |  |
|  | Yes | 221 | 69.1% | 215 | 69.4% | 6 | 60.0% | 2.7% | 0.66 | 0.18 - 2.40 | |  | - | - | | - |
|  |  |  |  |  |  |  |  |  |  |  | |  |  |  | |  |
|  | Drug use |  |  |  |  |  |  |  |  |  | |  |  |  | |  |
|  | No | 314 | 98.1% | 304 | 98.1% | 10 | 100.0% | 3.2% | - | - | | 1.000† | - | - | | - |
|  | Yes | 6 | 1.9% | 6 | 1.9% | 0 | 0.0% | 0.0% | - | - | |  | - | - | | - |
|  |  |  |  |  |  |  |  |  |  |  | |  |  |  | |  |
|  | Had a STI before |  |  |  |  |  |  |  |  |  | |  |  |  | |  |
|  | No | 263 | 82.2% | 258 | 83.2% | 5 | 50.0% | 1.9% | - | - | | **0.019†** | - | - | | - |
|  | Yes | 57 | 17.8% | 52 | 16.8% | 5 | 50.0% | 8.8% | 4.96 | 1.39 - 17.75 | |  | 4.11 | 1.12 - 15.10 | | **0.033** |
|  |  |  |  |  |  |  |  |  |  |  | |  |  |  | |  |
|  | * Likelihood Chi-squared test | |  |  |  |  |  |  |  |  | |  |  |  | |  |
|  | † Fisher's exact chi-squared test | |  |  |  |  |  |  |  |  | |  |  |  | |  |
