# Supplementary material for: Incidence of HIV and the Prevalence of HIV, Hepatitis B and Syphilis among Youths in Maputo, Mozambique: A Cohort Study
Source: PLoS One. 2015 Mar 23;10(3):e0121452. doi: 10.1371/journal.pone.0121452 (PMC4370560; doi:10.1371/journal.pone.0121452)
Supplement: S2 Table — (DOCX) [file pone.0121452.s002.docx]

| **S2 Table - Baseline socio-demographic and behavioral characteristics and HIV prevalence in female participants** | | | | | | | | | | |  |  |  |  | |
| --- | --- | --- | --- | --- | --- | --- | --- | --- | --- | --- | --- | --- | --- | --- | --- |
|  |  |  |  |  |  |  |  |  |  |  |  |  |  |  | |
|  | **Characteristic** | **Total** | | **HIV negative** | | **HIV positive** | | | **Unadjusted** | | | **Adjusted** | | | |
|  |  | **N** | **%** | **N** | **%** | **N** | **%** | **Prevalence** | **OR** | **CI 95%** | **p** | **OR** | **CI 95%** | | **p** |
|  | Total Screened | 1060 |  | 999 |  | 61 |  | 5.8% |  |  |  |  |  | |  |
|  |  |  |  |  |  |  |  |  |  |  |  |  |  | |  |
|  | Age (change in a year) | - | - | - | - | - | - | - | 1.27 | 1.09 - 1.48 | **0.002** | 1.37 | 1.18 - 1.59 | | **< 0.001** |
|  |  |  |  |  |  |  |  |  |  |  |  |  |  | |  |
|  | Marital Status |  |  |  |  |  |  |  |  |  |  |  |  | |  |
|  | Single | 1043 | 98.4% | 983 | 98.4% | 60 | 98.4% | 5.8% | - | - | 1.000† | - | - | | - |
|  | Married/Cohabitating | 17 | 1.6% | 16 | 1.6% | 1 | 1.6% | 5.9% | 1.02 | 0.13 - 7.85 |  | - | - | | - |
|  |  |  |  |  |  |  |  |  |  |  |  |  |  | |  |
|  | Education |  |  |  |  |  |  |  |  |  |  |  |  | |  |
|  | Primary and Secondary | 615 | 58.0% | 570 | 57.1% | 45 | 73.8% | 7.3% | - | - | **0.020†** | - | - | | - |
|  | Technical training | 260 | 24.5% | 253 | 25.3% | 7 | 11.5% | 2.7% | 0.35 | 0.16 - 0.79 |  | 0.31 | 0.14 - 0.71 | | **0.006** |
|  | University degree | 185 | 17.5% | 176 | 17.6% | 9 | 14.8% | 4.9% | 0.65 | 0.31 - 1.35 |  | 0.62 | 0.29 - 1.32 | | 0.214 |
|  |  |  |  |  |  |  |  |  |  |  |  |  |  | |  |
|  | Occupation |  |  |  |  |  |  |  |  |  |  |  |  | |  |
|  | Student | 1030 | 97.2% | 970 | 97.1% | 60 | 98.4% | 5.8% | - | - | 1.000† | - | - | | - |
|  | Employed | 30 | 2.8% | 29 | 2.9% | 1 | 1.6% | 3.3% | 0.56 | 0.07 - 4.16 |  | - | - | | - |
|  |  |  |  |  |  |  |  |  |  |  |  |  |  | |  |
|  | Religion |  |  |  |  |  |  |  |  |  |  |  |  | |  |
|  | Christian | 961 | 90.7% | 908 | 90.9% | 53 | 86.9% | 5.5% | 0.66 | 0.31 - 1.44 | 0.297† | - | - | | - |
|  | Other | 99 | 9.3% | 91 | 9.1% | 8 | 13.1% | 8.1% | - | - |  | - | - | | - |
|  |  |  |  |  |  |  |  |  |  |  |  |  |  | |  |
|  | Age at sexual debut (years) |  |  |  |  |  |  |  |  |  |  |  |  | |  |
|  | Less than 18 | 715 | 67.5% | 666 | 66.7% | 49 | 80.3% | 6.9% | - |  | **0.027** | - | - | | - |
|  | 18 or more | 345 | 32.5% | 333 | 33.3% | 12 | 19.7% | 3.5% | 0.49 | 0.26 - 0.93 |  | 0.39 | 0.20 - 0.76 | | **0.005** |
|  |  |  |  |  |  |  |  |  |  |  |  |  |  | |  |
|  | Number of sex partners in life |  |  |  |  |  |  |  |  |  |  |  |  | |  |
|  | 1 | 184 | 17.4% | 176 | 17.6% | 8 | 13.1% | 4.3% | - | - | 0.486† |  |  | |  |
|  | > 1 | 876 | 82.6% | 823 | 82.4% | 53 | 86.9% | 6.1% | 1.42 | 0.66 - 3.03 |  | - | - | | - |
|  |  |  |  |  |  |  |  |  |  |  |  |  |  | |  |
|  | Number of sex partners in the last 6 months |  |  |  |  |  |  |  |  |  |  |  |  | |  |
|  | 0 - 1 | 914 | 86.2% | 860 | 86.1% | 54 | 88.5% | 5.9% | - | - | 0.704† |  |  | |  |
|  | > 1 | 146 | 13.8% | 139 | 13.9% | 7 | 11.5% | 4.8% | 0.80 | 0.36 - 1.80 |  | - | - | | - |
|  |  |  |  |  |  |  |  |  |  |  |  |  |  | |  |
|  | Condom use in the last sexual intercourse |  |  |  |  |  |  |  |  |  |  |  |  | |  |
|  | No | 390 | 36.8% | 367 | 36.7% | 23 | 37.7% | 5.9% | - | - | 0.879 | - | - | | - |
|  | Yes | 670 | 63.2% | 632 | 63.3% | 38 | 62.3% | 5.7% | 0.96 | 0.56 - 1.64 |  | - | - | | - |
|  |  |  |  |  |  |  |  |  |  |  |  |  |  | |  |
|  | Alcohol consumption |  |  |  |  |  |  |  |  |  |  |  |  | |  |
|  | No | 593 | 55.9% | 564 | 56.5% | 29 | 47.5% | 4.9% | - | - | 0.173 | - | - | |  |
|  | Yes | 467 | 44.1% | 435 | 43.5% | 32 | 52.5% | 6.9% | 1.43 | 0.85 - 2.40 |  | - | - | | - |
|  |  |  |  |  |  |  |  |  |  |  |  |  |  | |  |
|  | Drug use |  |  |  |  |  |  |  |  |  |  |  |  | |  |
|  | No | 1059 | 99.9% | 998 | 99.9% | 61 | 100.0% | 5.8% | - | - | 1.000† | - | - | | - |
|  | Yes | 1 | 0.1% | 1 | 0.1% | 0 | 0.0% | 0.0% | - | - |  | - | - | | - |
|  |  |  |  |  |  |  |  |  |  |  |  |  |  | |  |
|  | Had a STI before |  |  |  |  |  |  |  |  |  |  |  |  | |  |
|  | No | 710 | 67.0% | 669 | 67.0% | 41 | 67.2% | 5.8% | - | - | 0.968 | - | - | | - |
|  | Yes | 350 | 33.0% | 330 | 33.0% | 20 | 32.8% | 5.7% | 0.99 | 0.57 - 1.71 |  | - | - | | - |
|  |  |  |  |  |  |  |  |  |  |  |  |  |  | |  |
|  | * Likelihood Chi-squared test | |  |  |  |  |  |  |  |  |  |  |  | |  |
|  | † Fisher's exact chi-squared test | |  |  |  |  |  |  |  |  |  |  |  | |  |
